# Integration of molecular typing results into tuberculosis surveillance in Germany—A pilot study

**DOI:** 10.1371/journal.pone.0188356

**Published:** 2017-11-22

**Authors:** Marta Andrés, Elke Göhring-Zwacka, Lena Fiebig, Martin Priwitzer, Elvira Richter, Sabine Rüsch-Gerdes, Walter Haas, Stefan Niemann, Bonita Brodhun

**Affiliations:** 1 Respiratory Infections Unit, Department for Infectious Disease Epidemiology, Robert Koch Institute, Berlin, Germany; 2 State Health Department (*Landesgesundheitsamt*) Baden-Württemberg, Stuttgart, Germany; 3 Local Public Health Office (*Gesundheitsamt*) Stuttgart, Stuttgart, Germany; 4 Labor Dr. Limbach, TB Laboratory, Heidelberg, Germany; 5 National Reference Center (NRC) for Mycobacteria, Research Center Borstel, Borstel, Germany; 6 Molecular and Experimental Mycobacteriology, Research Center Borstel, Leibniz-Center for Medicine and Biosciences, Borstel, Germany; 7 German Center for Infection Research, Partner Site Hamburg-Borstel-Lübeck, Borstel, Germany; Agencia de Salut Publica de Barcelona, SPAIN

## Abstract

An integrated molecular surveillance for tuberculosis (TB) improves the understanding of ongoing TB transmission by combining molecular typing and epidemiological data. However, the implementation of an integrated molecular surveillance for TB is complex and requires thoughtful consideration of feasibility, demand, public health benefits and legal issues. We aimed to pilot the integration of molecular typing results between 2008 and 2010 in the German Federal State of Baden-Württemberg (population 10.88 Million) as preparation for a nationwide implementation. Culture positive TB cases were typed by IS6110 DNA fingerprinting and results were integrated into routine notification data. Demographic and clinical characteristics of cases and clusters were described and new epidemiological links detected after integrating typing data were calculated. Furthermore, a cross-sectional survey was performed among local public health offices to evaluate their perception and experiences. Overall, typing results were available for 83% of notified culture positive TB cases, out of which 25% were clustered. Age <15 years (OR = 4.96, 95% CI: 1.69–14.55) and being born in Germany (OR = 2.01, 95% CI: 1.44–2.80) were associated with clustering. At cluster level, molecular typing information allowed the identification of previously unknown epidemiological links in 11% of the clusters. In 59% of the clusters it was not possible to identify any epidemiological link. Clusters extending over different counties were less likely to have epidemiological links identified among their cases (OR = 11.53, 95% CI: 3.48–98.23). The majority of local public health offices found molecular typing useful for their work. Our study illustrates the feasibility of integrating typing data into the German TB notification system and depicts its added public health value as complementary strategy in TB surveillance, especially to uncover transmission events among geographically separated TB patients. It also emphasizes that special efforts are required to strengthen the communication between local public health offices in different counties to enhance TB control.

## Introduction

Molecular typing of *Mycobacterium tuberculosis* (MTB) strains has proven to be a powerful tool for understanding MTB transmission [1–4]. From a surveillance perspective, molecular typing data provide high discriminatory information to uncover transmission events for tuberculosis (TB) cases that are bacteriologically confirmed. Usually, transmission is revealed by contact investigation, i.e. investigation of epidemiological links for active TB case finding. Contact investigation relies on the information on social contacts and whereabouts of patients and is subject to limitations, e.g. recall bias of short contact, mobility of patients, or reluctance to name possible contacts [5]. Yet epidemiological investigation, unlike molecular typing, can be performed even when no mycobacteria can be isolated from the patient [6] and it also encompasses latent TB infection (LTBI). Therefore, molecular typing and contact investigation are complementary approaches that can be combined to improve TB surveillance.

Germany is a low TB incidence country; however, in 2015, 5,865 cases (7.3 cases per 100 000 population) of active TB were reported [7], which was a considerable increase in comparison to previous years. This increase can be mainly attributed to a changing demographic context (migration and mobility) and it marks an end to the trend of declining TB notification rate in Germany for the last decade [8]. Currently, molecular typing results are not routinely used for TB control in Germany. Nonetheless, public health authorities, both in Germany and at the European level, identify the implementation of an integrated molecular surveillance (IMS) as a priority area to strengthen TB control [9].

The implementation of a TB integrated molecular surveillance (IMS) is a complex public health intervention and requires careful planning to ensure that all technical, legal, and practical demands are addressed and that an added public health benefit is obtained [10, 11]. Thus, in this study, we piloted the feasibility and yield of prospective integration of molecular typing data of bacteriologically confirmed TB cases from the German Federal State of Baden-Württemberg into the German TB notification system. We also aimed to evaluate the collected data to describe the identified clusters of TB cases and to assess the added value attributable to the molecular typing information.

## Materials and methods

### Study population

The pilot study was performed between January 2008 and December 2010 in Baden-Württemberg, the third largest German Federal State (population 10.88 Million). The study population included all notified TB cases that fulfilled the reference definition [12] and were bacteriologically confirmed. Cases were notified by physicians and laboratories to local public health offices in accordance with the German Protection Against Infection Act (IfSG) and then transmitted anonymously via the state health department to the Robert Koch Institute (RKI), Germany’s national public health institute.

### Data sources

#### TB notification data

Anonymous case-based TB notification data from RKI’s notifiable diseases database, SurvNet@RKI, were used (export date: 30 August 2011). The key variables included age, sex, country of birth, and multidrug resistance status [13].

#### Molecular typing data

Molecular typing of TB isolates was performed in the National Reference Laboratory (NRL) for Mycobacteria at the Research Center Borstel upon request from local public health offices. Mycobacterial DNA extraction and DNA fingerprinting, using IS*6110* as a probe, were performed according to a standardized protocol [14]. Additionally, all isolates were analyzed by spoligotyping [15]. Molecular typing data were analyzed with Bionumerics 7.5 software (Applied Maths, Sint-Martens-Latem, Belgium). Spoligotyping data were used to additionally confirm strain relationships and for genotypic classification in accordance with the MIRU-VNTRplus database [16, 17]. Patients with mixed patterns indicating a double infection with two MTB strains were excluded from further analysis as no clear IS*6110* band definition was possible in such mixed-strain isolates. Clusters of strains with less than five IS6110 bands were analyzed by 24-locus Mycobacterial Interspersed Repetitive Unit–Variable Number of Tandem Repeats (24-locus MIRU-VNTR) typing using the MIRU-VNTR typing kits (Genoscreen, Lille, France) as described previously [18].

### Data management

#### Molecular typing data management

**Fig 1:**
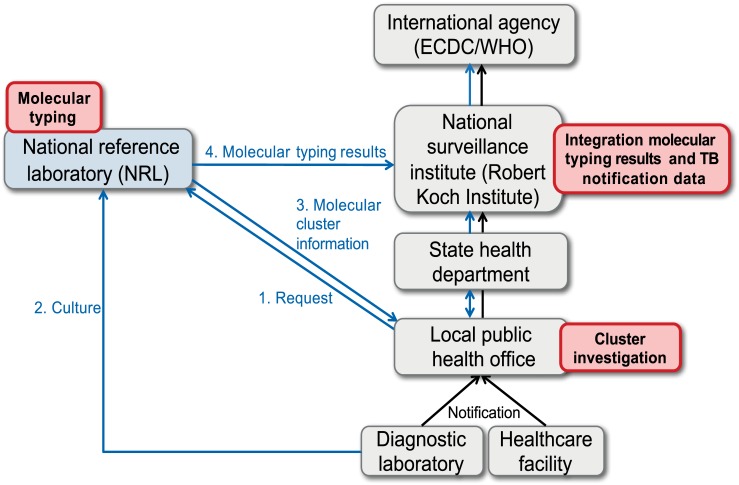
Pilot data flow of the molecular typing results in Baden Württemberg (Germany). 1. Molecular typing requested by local public health offices to NRL; 2. Culture shipment to NRL; 3. Communication of molecular cluster information from NRL to local public health offices and 4. Communication of full molecular typing results from NRL to RKI.

Each TB case had a unique reference number allocated by the local public health office. The offices sent an official request to the NRL to perform the molecular typing. According to the typing results, the NRL allocated TB cases to clusters if the same molecular pattern was shared. The NRL reported the following data back to the local public health office: laboratory identifier, cluster status (yes/no), and, if applicable, also cluster number. Furthermore, if no molecular typing results were available (e.g. missing culture), this was also reported. The local public health office electronically transmitted this information, once anonymized, together with case-based routine TB notification data (see above) via the state health department of Baden-Württemberg to the RKI. Before transmitting the information to the RKI, the state health department checked whether clusters contained cases in different counties (administrative districts). If this was the case, the involved public health offices were informed to initiate further epidemiological investigations (Fig 1).

Moreover, upon receiving a report of a clustered case, the local public health offices checked the epidemiological information available for other cases in the cluster. If appropriate, they initiated additional epidemiological investigations. Molecular clusters were categorized depending on identified epidemiological links (see below). This information was also transmitted as a notification update to the RKI via the state health department.

#### Data linkage

At RKI, a new database was manually created to link the TB notification data (including the molecular cluster information) with the full molecular typing data provided by the NRL directly to RKI. Both sources could be linked using the unique reference number. The molecular typing data included DNA fingerprinting and spoligotyping results.

### Cluster definition and categorization

A molecular cluster was defined as a group of TB patients sharing identical IS6110 and spoligotyping patterns. For clusters with strains having less than five IS6110 bands, identity was confirmed by MIRU-VNTR typing. Molecular clusters were grouped by local public health offices into four categories according to the evidence of epidemiological links among their cases: (i) “New epi links”, molecular typing information led to the uncovering of previously unknown epidemiological links; (ii) “Epi links previously known”, the existence of epidemiological links was already known and the molecular typing results confirmed them; (iii) “Epi links partially known”, the existence of epidemiological links was previously partially known through routine contact investigation, but the molecular typing results led to the identification of new members; (iv) “No epi links”, no epidemiological link could be found among the TB patients even after typing results were available.

### Statistical analysis

Demographic and clinical characteristics of cases and clusters were described in terms of numbers and percentages. Chi-squared test was used to assess differences in categorical variables. Stratified analyses and multivariable logistic regression analyses were performed to examine the association between (i) being part of a molecular cluster and demographic and clinic characteristics, including age, sex, country of birth, multidrug-resistant (MDR)-TB, and MTB strain, ii) belonging to a specific cluster category and cluster characteristics, including size, geographical extent and patients´ country of birth (only Germany, only other, and mixed cluster) and iii) belonging to “No epi links” and demographic characteristics of the clustered TB patients, including age, sex and country of birth. Based on the regression coefficient of the model, odds ratio (OR) with their 95% confidence interval (CI) were calculated to assess the strength of association. Data were processed and analyzed in Stata/SE 14.1 (Stata corporation, Texas, USA).

### Survey

A cross-sectional questionnaire survey was performed in 2011 by the state health department of Baden-Württemberg among 38 participating local public health offices to evaluate the performance of the pilot study and to assess their perception of using molecular typing in their daily work (S1 and S2 Appendices).

### Legal basis, data protection and ethics

Anonymized notification data and results of molecular typing were received within the legal framework of the German Protection Against Infection Act (IfSG, *Infektionsschutzgesetz*). The questionnaires were answered by the local Health Authorities and did not contain personal information. As no interventions were performed and only fully anonymized data were analysed no further ethical clearance was required.

## Results

### Characteristics of clustered and non-clustered cases

From 2008 to 2010, 1,620 TB cases were reported in the federal state of Baden-Württemberg in Germany, of which 69% (1,111/1,620) were bacteriologically confirmed. Molecular typing could be performed in 83% (918/1,111) of the culture positive cases. Of these 918 cases, 25% (226/918) were clustered by molecular typing and 75% (692/918) were not clustered (Table 1). The proportion of clustered cases was largely stable over time (24%, 74/312 in 2008; 24%, 75/317 in 2009 and 27%, 77/289 in 2010).

**Table 1:** Characteristics of tuberculosis cases with molecular typing results in Baden-Württemberg (Germany).

Demographic features of TB cases	Total	Clustered	Non-clustered	Factors associated with clustering, Multivariable analysis	
				aOR (95% CI)	p-value
Number of TB cases	918	226 (24.6%)	692 (75.4%)		
Age					
Age median (IQR)	51 (33–69)	45 (30–58)	53 (34.5–71)		
<15 years	18	12 (66.7%)	6 (33.3%)	4.96 (1.69–14.55)	**0.004**
≥ 15–59 years	571	162 (28.4%)	409 (71.6%)	1	
≥ 60 years	329	52 (15.8%)	277 (84.2%)	0.39 (0.27–0.57)	**< 0.001**
Sex					
Male	520	136 (26.2%)	384 (73.8%)	1.10 (0.79–1.53)	0.554
Female	398	90 (22.6%)	308 (77.4%)	1	
Origin					
German-born	389	119 (30.6%)	270 (69.4%)	2.01 (1.44–2.80)	**< 0.001**
Foreign-born	496	100 (20.2%)	396 (79.8%)	1	
Drug resistance					
MDR	10	3 (30.0%)	7 (70.0%)	1.65 (0.4–6.86)	0.490
Non-MDR	820	204 (24.9%)	616 (75.1%)	1	
Strain					
Harleem	288	82 (28.5%)	206 (71.5%)	1	
LAM	67	12 (17.9%)	55 (82.1%)	0.54 (0.26–1.09)	0.087
Beijing	56	24 (42.9%)	32 (57.1%)	1.81 (0.93–3.53)	0.081
Delhi	44	2 (4.5%)	42 (95.5%)	0.40 (0.17–0.98)	**0.046**
EAI	49	7 (14.3%)	42 (85.7%)	0.53 (0.26–1.09)	0.087

Data indicate number of TB cases and (percent), except for age where median and (interquartile range) are shown. aOR = adjusted odds ratio; EAI: East African-Indian; LAM: Latin American-Mediterranean; MDR: multidrug-resistant.

**Fig 2:**
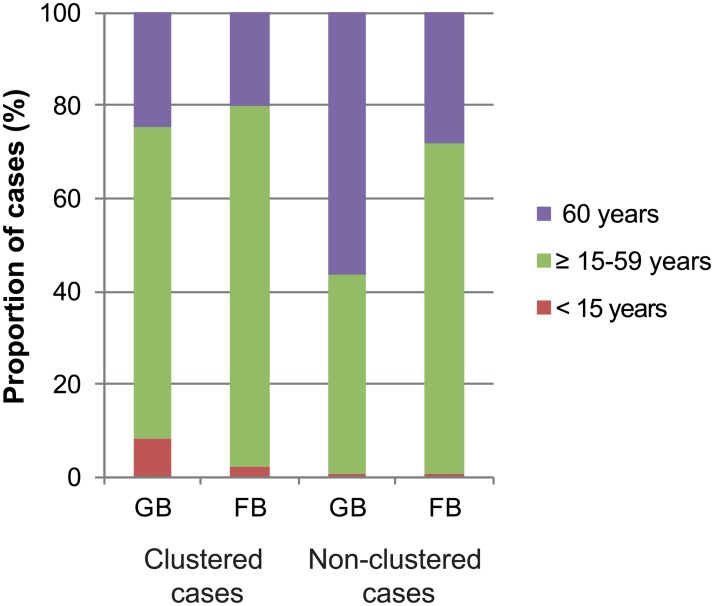
Age distribution of clustered and non-clustered TB cases in Baden-Württemberg (Germany), categorized according to origin. GB: German-born; FB: foreign-born.

TB cases belonging to a molecular cluster were younger than non-clustered cases (median 45 years, interquartile range (IQR) 30–58 vs 53 years, IQR 34.5–71, respectively). Clustered patients were mostly German-born (54%) while non-clustered patients were mostly foreign-born (59%). Among non-clustered cases, the age distribution was different between German-born and foreign-born patients (64 years, IQR 51–71 vs 41 years, IQR 30–62.5; p<0.001, Fig 2). Sex was not significantly different between both groups (60% males among clustered cases vs 55% males among non-clustered cases). The proportion of MDR-TB cases was similar among clustered and non-clustered groups (1.5% vs 1.1%, respectively). Around 60% (552/918) of the strains investigated could be assigned to a specific phylogenetic lineage of the MTB complex. Haarlem was the most common strain type both among clustered and non-clustered patients (Table 1).

In multivariable analysis, the odds ratio for being clustered was 5.0 (95%CI: 1.69–14.55, p = 0.004) for cases aged <15 years and 0.39 (95%CI: 0.27–0.57, p<0.001) for cases aged >60 years compared to cases aged ≥15–59 years. The geographical origin was also significantly associated with clustering, the odds ratio for being clustered was 0.49 for foreign-born cases in comparison to German-born cases (95%CI: 0.36–0.69, p<0.001; Table 1).

### Characteristics of molecular clusters

The 226 clustered TB cases were distributed in 85 molecular clusters. The median cluster size was 2 (IQR 2–3) and most clusters (93%, 79/85) comprised of <5 cases. In addition, 5.9% (5/85) of clusters were medium in size (5–9 cases) and one cluster comprised of 10 cases.

**Fig 3:**
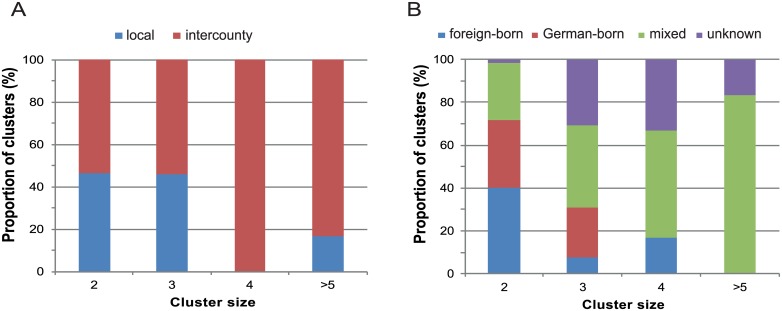
Molecular cluster characteristics in Baden-Württemberg (Germany) by A. Geographical distribution and B. Patient origin. Number of clusters; Size 2 (n = 60); Size 3 (n = 13); Size 4 (n = 6); Size >5 (n = 6).

Concerning the geographical distribution, 59% of the clusters spanned over more than one county, here referred to as “intercounty” (originally notified from different local public health offices) and 41% were local (belonging to a single county, i.e. the same local public health office) (Fig 3A). All clusters larger than three patients were intercounty, except for one cluster with 8 cases that was local. Considering patient country of birth, most clusters were mixed, with both German-born and foreign-born patients (34%); followed by clusters with only foreign-born patients (31%) and clusters with only German-born patients (26%). Of those clusters with only two cases, 40% contained only foreign-born patients, while 32% comprised of only German-born patients, and 27% were mixed. (Fig 3B). In this regard, it is important to underline that different countries of birth do not preclude family relations.

### Epidemiological information for molecular clusters (cluster-level analysis)

Seventy molecular clusters (82%, 70/85) were grouped according to the epidemiological links among their cases (for the remaining 15 clusters this information was not reported, see Methods). The majority of molecular clusters were categorized as “No epi links” (59%) since an epidemiological link was neither detected before nor after receiving typing results. The second most common category was “Epi links previously known” comprising 17% of the molecular clusters; followed by “Epi links partially known” (13%) and “New epi links” (11%, Table 2). Intercounty molecular clusters were mostly “No epi links” (75% compared to 31% of local molecular clusters). Local clusters were mostly “Epi links previously known” (42% compared to 2.3% of intercounty clusters, Table 2). The “No epi links” category was also the most common among molecular clusters irrespective of country of birth (71% of foreign-born, 53% of German-born and 52% of mixed clusters, Table 2).

**Table 2:** Cluster categories combining molecular and epidemiological information and cluster level analysis in Baden-Württemberg (Germany).

Demographic features of cluster	Total	“New epi links”	“Epi links previously known”	“No epi links”	“Epi links partially known”
Number of clusters	70	8 (11.4%)	12 (17.1%)	41 (58.6%)	9 (12.8%)
Size					
Median size (IQR)	2 (2–3)	2 (2–2)	2 (2–3)	2 (2–2)	5 (3–6)
2 TB cases	49	7 (14.3%)	8 (16.3%)	34 (69.4%)	0
3–5 TB cases	18	1 (5.6%)	4 (22.2%)	7 (38.9%)	6 (33.3%)
>5 TB cases	3	0	0	0	3 (100%)
Geographical extent					
Local	26	5 (19.2%)	11 (42.3%)	8 (30.8%)	2 (7.7%)
Intercounty	44	3 (6.8%)	1 (2.3%)	33 (75%)	7 (15.9%)
Origin					
Only German-born	17	2 (11.8%)	5 (29.4%)	9 (52.9%)	1 (5.9%)
Only foreign-born	21	3 (14.3%)	3 (14.3%)	15 (71.4%)	0
mixed	25	3 (12.0%)	2 (8.0%)	13 (52.0%)	7 (28.0%)

Data indicate number of clusters and (percent), except for size where median and (interquartile range) are shown.

An association was found between “No epi links” and intercounty distribution of the cluster (OR 11.53, 95% CI: 3.48–38.23, p<0.001). Furthermore, local clusters had an odds ratio of 22.46 (95% CI: 2.74–184.23, p = 0.004) for belonging to “Epi link previously known” in comparison to clusters in other categories.

### Epidemiological information for patients within molecular clusters (case-level analysis)

“No epi links” comprised the highest proportion of patients (49%) followed by “Epi links partially known” (25%), “Epi links previously known” (16%) and “New epi links” (9.6%). The age distribution was different between these categories (p = 0.001); TB patients belonging to “Epi links previously known” were the youngest (35 years, IQR 18–45), and those belonging to “No epi links” the oldest (49 years, IQR 34–66, Table 3).

**Table 3:** Demographic data of TB cases belonging to different clusters categories in Baden-Württemberg (Germany).

Demographic features of TB cases	Total	“New epi links”	“Epi links previously known”	“No epi links”	“Epi links partially known”
TB cases	187	18 (9.6%)	30 (16.1%)	92 (49.2%)	47 (25.1%)
Age					
Age median (IQR)	45 (30–58)	41.5 (24–56)	35 (18–45)	49 (34–66)	46 (27–59)
<15 years	11	0	5 (45.5%)	0	6 (54.5%)
≥ 15–59 years	134	16 (12%)	22 (16.4%)	65 (48.5%)	31 (23.1%)
≥ 60 years	41	2 (4.9%)	2 (4.9%)	27 (65.9%)	10 (24.3%)
Sex					
Male	112	13 (11.6%)	16 (14.3%)	52 (46.4%)	31 (27.7%)
Female	74	5 (6.7%)	13 (17.6%)	40 (54.1%)	16 (21.6%)
Origin					
German-born	94	9 (9.6%)	19 (20.2%)	34 (36.1%)	32 (34.1%)
Foreign-born	86	9 (10.5%)	8 (9.3%)	55 (63.9%)	14 (16.3%)

Data indicate number of TB cases and (percent), except for age where median and (interquartile range) are shown.

Multivariable logistic regression revealed that foreign-born (OR 3.01, 95% CI: 1.54–5.91, p<0.001) and age > 60 years (OR 2.42, 95% CI: 1.5–5.3, p = 0.028) were associated with being part of a “No epi links” cluster (Table 4).

**Table 4:** Factors associated with TB patients in “No epi links” clusters compared to patients in all other cluster categories in Baden-Württemberg (Germany).

Demographic features of TB cases	Clustered cases in “No epi links”	Clustered cases in other cluster categories	Multivariable analysis	
			aOR (95% CI)	p-value
TB cases	92 (49.2%)	95 (50.8%)		
Age				
Age median (IQR)	49 (34–66)	37.5 (25–55)		
<15 years	0	11 (100%)	undefined	
≥ 15–59 years	65 (48.5%)	69 (51.5%)	1	**0.028**
≥ 60 years	27 (65.9%)	14 (34.1%)	2.42 (1.1–5.3)	
Sex				
Male	52 (46.4%)	60 (53.6%)	1 (0.5–2)	0.991
Female	40 (54.1%)	34 (45.9%)	1	
Origin				
German-born	34 (36.2%)	60 (63.8%)	1	**0.001**
Foreign-born	55 (64%)	31 (36%)	3.01 (1.54–5.91)	

Data indicate number of TB cases and (percent), except for age where median and (interquartile range) are shown. aOR = adjusted odds ratio.

### Engagement and benefits perceived by local public health offices

All 38 local public health offices in Baden-Württemberg, all with at least one notified TB case, participated in the study, each reporting a median of 29 TB cases (range 8 to 150 cases), a median of 20 culture positive TB cases (range 5 to 116) and a median of 17 molecularly typed TB cases (range 2 to 98 cases). More than 71% of the local public health offices reported molecular data for at least 75% of the culture positive TB cases.

Overall, 97% (37/38) of local public health offices answered the questionnaire. A majority found the molecular typing information useful for their work (73%, 27/37) and 57% (21/37) indicated that molecular typing information influenced contact investigations, mostly by involving other local public health offices (95%, 20/21), but also by expanding the contact investigation (24%, 5/21) or prolonging the clinical monitoring of contact persons and persons with LTBI (19%, 4/21). One local public health office (4.7%, 1/21) reported having initiated a new contact investigation based on typing information. Overall, 45% of local public health offices (14/31) answered that epidemiological investigations were always initiated upon receiving the typing results if epidemiological links were not previously known between molecularly linked cases; 35% (11/31) stated that they were sometimes initiated and 19% (11/31) never initiated such investigations. Most (84%, 26/31) local public health offices reported that it was not possible to detect new epidemiological links for TB cases linked molecularly.

## Discussion

In this study, we piloted the integration of molecular typing results into TB notification data between 2008 and 2010 in the German Federal State of Baden-Württemberg. Our study showed the feasibility of this measure under the current German legal framework. Collected data reveal that younger age and German origin were factors associated with TB clustering in this setting. The study also indicated an added public health value of typing data to uncover new epidemiological links not detected by conventional epidemiological investigations and highlighted the limits of epidemiological investigation when patients are geographically dispersed. Moreover, our study showed that a number of local public health offices benefited from utilizing molecular typing results in their daily TB control activities, indicating the positive aspects of such results.

The proportion of clustered cases in our study (25%) was within the range of previous reports that used molecular typing in low TB incidence settings; such as Finland (34%) [19], Slovenia (31%) [20], Sweden (21%) [21], UK (57%) [22], and the city of Hamburg in Germany (34%) [4]. The study periods in these works were variable, likely leading to the differences in clustering, as longer periods usually increase clustering rates. Interestingly, an earlier study in Baden-Württemberg between 2003 and 2005 using IS6110 DNA fingerprinting as the typing method reported a lower clustering rate (17%) than in our study [23].

In our setting, demographic characteristics differed between clustered and non-clustered patients, clustered cases being younger and mostly German-born (Table 1). As shown in other studies [1, 2, 24], clustering rates decreased with older age. Children aged <15 years were more often clustered (66%) than adults 15–59 years (28%), and adults ≥60 years were rarely clustered (16%); this is consistent with TB in children as it points towards recent transmission and, therefore, higher clustering, whereas TB in the elderly represents reactivation of TB acquired many years prior [25]. Regarding the origin of patients, those born outside of Germany were less often clustered, likely explained by the fact that TB in immigrants is to a great extent caused by reactivation of latent TB acquired in the country of birth [1, 2, 24, 26, 27]. However, it is also likely that the high mobility of immigrants hinders cluster detection since our study was restricted to a single German federal state.

In eight (11%) molecular clusters, epidemiological links were identified after integrating molecular typing information. This understates the added public health benefit of molecular typing and its complementary value to conventional epidemiological investigation. In a study in the Netherlands, the proportion of clustered cases in which new epidemiological links were discovered with typing information was 24% [26]. The higher rate in the Dutch study could be due to the longer study period (6 years). Additionally, 13% of the molecular clusters in our study fell in the category “Epi links partially known”, where new TB cases were added to partially known clusters. Moreover, in 17% of clusters, all epidemiological links among their members were known before typing information confirmed the transmission. TB patients belonging to this category were younger in comparison to the rest of clustered TB cases, likely due to the shorter time between TB infection and disease onset in children. Combining the last two categories shows that only 30% of the TB transmission clusters were known before cluster feedback, once again highlighting the importance of molecular typing as a complementary strategy in TB control.

The last category, “No epi links”, which includes clusters in which no epidemiological links could be identified even after cluster feedback, was the most common (59% of clusters; Table 2), as reported in other studies as well [3, 4, 26, 28]. In our study, most “No epi links” (70%) clusters contained only two TB cases. Two associated factors stand out in this category: i) older age, since TB transmission likely took place in the past; ii) geographical dispersion of cases (75% of intercounty clusters belonged to this category). This finding indicates that epidemiological cluster investigations in our study setting appears to be limited to the county level. Considering that in our study 60% of the molecular clusters were intercounty (e.g. the associated cases were located in different local counties), an extra effort is needed to ensure that communication between local public health offices in different administrative counties is improved. A third factor that was associated with the “No epi links” category was being born outside of Germany (Table 4), possibly due to difficulties associated with reaching this population group for contact investigations. However, it can be argued whether molecular clustering in foreign-born cases actually represents recent domestic transmission or false clustering due to TB imported from countries of origin with circulation of highly genetically-related MTB strains [23]. A recent study shows that clusters identified using 24-locus MIRU-VNTR typing among immigrants in a low-incidence setting were overestimated after clusters were revisited using whole genome sequencing (WGS)-based typing [29]. Ideally, the use of WGS in our study would have allowed us to discern whether the “No epi links” clusters represented real TB transmission events or, to some extent, clustering overestimation [30, 31]. WGS is arising as a powerful tool to study TB transmission due to its sensitivity to determine the timing and direction of transmission [32–34] and presently the shift towards using WGS as a molecular typing methodology to substitute classical typing methods is in progress in different countries [22, 35], although standardization and cost-effectiveness issues still need to be addressed [30].

Engagement with the local public health offices was essential for this study. The cross-sectional survey showed that most local public health offices found molecular typing useful for their work and slightly more than half indicated that it had an influence in the way they performed contact investigations by involving other local public health offices and by expanding the investigations. These results indicate a need for the implementation of IMS and that it would be endorsed by the local public health offices in Germany. Similar attitudes were reported in another study about strain typing services in UK [10].

### Limitations

This study is subject to several limitations. To comply with the format of the German TB notification data, it was not possible to identify individual epidemiological case-to-case links that were newly identified after cluster feedback. Rather, the information collected identified the clusters as a unit and showed whether epidemiological links were identified within the cluster as a whole. Therefore, we cannot evaluate how often molecular typing ruled out false epidemiological links. Regarding the origin of patients, it should be noted that information about the origin of parents was not collected in the TB notification data and, hence, German-born children can also be born to immigrants not born in Germany and comprise a mixed cluster containing both German-born and foreign-born patients [23]. Moreover, the short duration of the study (three years) and the use of IS6110 DNA fingerprinting as typing methodology, which does not have enough discriminatory power to distinguish single transmission events, hampered an in-depth understanding of MTB transmission dynamics. Another limitation was that manual data management steps were involved (e.g. cluster detection and linkage of molecular typing information and notification data); these are time-consuming and can be prone to human error, although a quality assurance analysis was undertaken. Furthermore, the timespan between finding a TB case at the local public health office and receiving the typing results was long. This might have reduced the impact of the typing data on the contact investigations. More automatized solutions would be needed to scale-up and accelerate this process.

## Conclusions

In conclusion, our pilot study demonstrated the feasibility of integrating molecular typing results of MTB isolates into the routine, ongoing German TB surveillance system in terms of alignment with the current legal framework (the Protection Against Infection Act), achievement of high coverage, and positive perception by local public health offices. Regarding the public health benefits, molecular typing data contributed to the identification of new epidemiological links. However, our study showed that for most molecularly related cases, it was not possible to establish epidemiological links, especially when different local public health offices were involved. This highlights the importance of molecular typing as complementary strategy to epidemiological investigation and emphasizes that special efforts are required to strengthen the communication between local public health offices in different counties in order to enhance TB control practice in Germany.

## Supporting information

S1 Appendix Survey to local public health offices (English). Survey on the project for the molecular typing of tuberculosis cultures in Baden-Württemberg from 2008 to 2010.
(DOC)

S2 Appendix Survey to local public health offices (German). Fragebogen zum Typisierungsprojekt der Tuberkulose-Kulturen in Baden-Württemberg 2008–2010.
(DOC)
