# Supplementary material for: Integration of molecular typing results into tuberculosis surveillance in Germany—A pilot study
Source: PLoS One. 2017 Nov 22;12(11):e0188356. doi: 10.1371/journal.pone.0188356 (PMC5699808; doi:10.1371/journal.pone.0188356)
Supplement: S1 Appendix — Survey on the project for the molecular typing of tuberculosis cultures in Baden-Württemberg from 2008 to 2010. (DOC) [file pone.0188356.s001.doc]

**S1 Appendix. Survey to local public health offices (English)**

**Survey on the project for the molecular typing of tuberculosis cultures in**

**Baden-Württemberg from 2008 to 2010**

1. How many molecular clusters did your local public health office detected from 2008 to 2010?

No clusters (Please, go to question 5)

fewer than 3

3 clusters or more

1. Were additional measures undertaken or the course of action during contact investigations influenced by the molecular cluster information?

yes  no  I do not know

If yes, in which way?

Initiation of contact investigation

Expansion of contact investigation

Inclusion of other local public health office in the contact investigation

Prolongation of the clinical monitoring of contact persons and persons with LTBI

Other (Please, describe)

1. If the typing results from Borstel showed that some TB cases belonged to a molecular cluster but no epidemiological links were known among these cases, was an investigation initiated?

always

sometimes

never (Please, go to question 5)

1. Was it possible, based on the typing information and the derived investigations, to identify new epidemiological links that were previously unknown (i.e. molecular cluster without epidemiological links previously known among their cases)?

yes, in all cases

in more than half of the cases

in less than half of the cases

no, in none of the cases

1. Do you find the molecular cluster information provided by the NRZ useful for your work?

yes  no  I do not know

1. Please, let us know if you have any other comment:

**Thank you very much for your support!**
