# Supplementary material for: Integration of molecular typing results into tuberculosis surveillance in Germany—A pilot study
Source: PLoS One. 2017 Nov 22;12(11):e0188356. doi: 10.1371/journal.pone.0188356 (PMC5699808; doi:10.1371/journal.pone.0188356)
Supplement: S2 Appendix — Fragebogen zum Typisierungsprojekt der Tuberkulose-Kulturen in Baden-Württemberg 2008–2010. (DOC) [file pone.0188356.s002.doc]

**S1 Appendix. Survey to local public health offices (German)**

**Fragebogen zum Typisierungsprojekt der Tuberkulose-Kulturen**

**in Baden-Württemberg 2008 – 2010**

1. Von wie vielen **molekularbiologischen Clustern** in 2008 – 2010 war ihr Gesundheitsamt betroffen?

von keinem Cluster => Bitte weiter mit Frage 5

weniger als 3 Cluster

3 Cluster oder mehr

1. Wurden auf Grundlage der molekularbiologischen Cluster-Informationen zusätzliche Maßnahmen veranlasst bzw. die Vorgehensweise bei Umgebungsuntersuchungen relevant beeinflusst/geändert?

ja  nein  weiß nicht

Wenn ja – in welcher Weise?

Initiierung einer Umgebungsuntersuchung

Ausweitung einer bereits durchgeführten Umgebungsuntersuchung

Einbeziehung anderer am Cluster beteiligter Gesundheitsämter

Verlängerung der Überwachungsdauer der ermittelten Kontaktpersonen/latent Infizierten

Sonstiges => Bitte kurz beschreiben

1. Wenn **kein epidemiologischer Zusammenhang der Fälle bekannt war**, die aufgrund der Typisierung in Borstel aber einem molekularbiologischen Cluster angehörten, wurden dann Ermittlungen auf Grund der Typisierungsergebnisse eingeleitet?

immer

zum Teil

nie => Bitte weiter mit Frage 5

1. Konnten nachträglich in diesen Fällen (kein epidemiologischer Zusammenhang jedoch molekularbiologischer Cluster [siehe Frage 3]) durch die molekularbiologischen Cluster-Informationen und die daraufhin eingeleiteten Nachermittlungen in Ihrem Gesundheitsamt epidemiologische Zusammenhänge festgestellt werden, die Ihnen vorher noch nicht bekannt waren?

ja, bei allen Fällen

in mehr als der Hälfte der Fälle

in weniger als der Hälfte der Fälle

nein, bei keinem der Fälle

1. Finden Sie die molekularbiologischen Cluster-Informationen des NRZ insgesamt hilfreich für Ihre Arbeit?

ja  nein  weiß nicht

1. Weitere Anregungen/Hinweise von Ihrer Seite:

**Vielen Dank für Ihre Unterstützung!**
